# Synthesis and characterization of a silver nanoparticle-containing polymer composite with antimicrobial abilities for application in prosthetic and orthotic devices

**DOI:** 10.1186/s40824-020-00191-6

**Published:** 2020-08-12

**Authors:** Catalina Quintero-Quiroz, Luz E. Botero, Diana Zárate-Triviño, Natalia Acevedo-Yepes, Jorge Saldarriaga Escobar, Vera Z. Pérez, Luis Javier Cruz Riano

**Affiliations:** 1 Centro de Bioingeniería, Grupo de investigaciones en Bioingeniería, Universidad Pontificia Bolivariana, circular 1 No. 73-76, Medellín, 050031 Colombia; 2 Grupo de Investigación de Biología de Sistemas,Universidad Pontificia Bolivariana, Cl 78B No. 72A-109, Medellín, 050031 Colombia; 3 Laboratorio de Inmunología y Virología, Universidad Autónoma de Nuevo León, Ave. Pedro de Alba S/N Ciudad Universitaria San Nicolás de los Garza, Monterrey, 64450 México; 4 Grupo de Investigación Sobre Nuevos Materiales, Universidad Pontificia Bolivariana, Cq.1 No. 70-01, Medellín, 050031 Colombia; 5 Facultad de Ingeniería Eléctrica y Electrónica, Cq.1 No. 70-01, Medellín, 050031 Colombia

**Keywords:** Synthesis, Silver nanoparticles, Silicon elastomer, Interface, Antimicrobial activity, Prosthesis, Characterization

## Abstract

**Background:**

The presence of skin problems in patients using external lower limb prosthesis is recurrent. This has generated the need to develop interfaces for prosthesis with the ability to control microbial growth. Silver nanoparticles (AgNPs) have been implemented in the development of biomaterials because of their high antimicrobial activity. This article discusses the development of an AgNP-containing polymer composite with antimicrobial activity for developing prosthetic liners.

**Methods:**

AgNPs were synthesized using a photochemical method and certain physicochemical properties were characterized. Furthermore, the antimicrobial activity of AgNPs against *Staphylococcus aureus* ATCC 25923 and methicillin-resistant *Staphylococcus aureus* (MRSA), was assessed on the basis of their minimum inhibitory concentrations (MICs). AgNPs were incorporated into a silicon elastomer to assess certain physicomechanical properties, antimicrobial activity and cytotoxic effect of the material.

**Results:**

The maximum antimicrobial activity of the material against *Staphylococcus aureus* ATCC 25923 and MRSA was 41.58% ±2.97% at AgNP concentration of 32.98 *μ*g/mL and 14.85% ±5.94% at AgNP concentration of 16.49 *μ*g/mL, respectively. Additionally, the material exhibited tensile yield strength, rupture tensile strength, and tensile modulus of elasticity of 0.70 - 1.10 MPa, 0.71–1.06 MPa, and 0.20 - 0.30 MPa, respectively. The mechanical characteristics of the material were within the acceptable range for use in external lower limb prosthetic and orthotic interfaces.

**Conclusions:**

It was possible to incorporate the AgNPs in a silicone elastomer, finding that the composite developed presented antimicrobial activity against *Staphylococcus aureus* ATCC 25923 and MRSA when compared to non-AgNP material samples.

## Background

The use of external prosthesis represents a practical alternative for modifying the structural or functional characteristics of the neuro–muscular–skeletal system of a person who has suffered limb loss [1]. As prosthesis use requires permanent contact with limb skin for an extended period, it is estimated that between 32% and 90.90% of the population who wear a lower limb prostheses usually reports at least one problem in the skin that is in contact with the device [2–8]

In fact, the heat generated by the contact socket, skin transpiration, and humidity conditions, together with mechanically generated skin disorders, favor bacterial growth [5, 9]. The microorganisms found in the skin of people wearing prostheses include *Staphylococcus aureus* and methicillin-resistant *Staphylococcus aureus* (MRSA), which are the most common cause of cutaneous infections [10]. These Gram positive bacteria can cause abscess, botryomycosis, carbunculosis, cellulitis, ecthyma, fascitis, foliculitis, furuncles, impetico, necrotizing, pyomyositis, and scalded-skin syndrome, among other infections [10, 11]. Skin infections are more frequent in immunocompromised patients, and given the presence of microorganisms, other preexisting skin disorders may reappear [12]. These infections may be hard to eradicate; therefore, most users decide to restrict the use of prostheses [13, 14].

Currently, some nanoscale materials have emerged as new antimicrobial agents to substitute common antibiotics in certain applications [15]. Silver nanoparticles (AgNPs) shows strong antimicrobial activity and have been proven to interrupt biofilms formed by bacterial agglomeration and their adherence to solid surfaces [16].

Three possible AgNPs toxicity mechanisms have been recently studied. The first one establishes that electrostatic interactions enable the union of positive Ag ions with the cell membrane of negatively charged microorganisms, generating lysis [17, 18]. Likewise, AgNPs have been linked to the interruption of the respiratory chain of cellular mitochondria and the synthesis of adenosine triphosphate (ATP) [15, 17, 18]. Finally, AgNPs may affect bacterial DNA and replication [18]. The antimicrobial activity of AgNPs depends on their size, shape, distribution and concentration, in addition to the microorganism species [16, 18]. However, some studies have reported that at low concentrations, AgNPs are not toxic to human beings given that these NPs actively interact through peptidoglycans that are only present on the cell wall of bacteria [12, 16, 19].

This way, AgNPs have been incorporated into polymers, where these can act as deposits of Ag ions, which are released to the medium or act upon contact [20–24]. Lower limb prostheses have liners, i.e., systems designed to achieve an adequate adjustment with the stump [25–27]. Liners are commonly manufactured using silicon elastomers since they are highly reticulated, of viscoelastic nature and grant comfort, are soft to the touch, and have good impact absorption [25, 28].

In this study, a composite material with ability to control bacterial proliferation was developed for application as the interface for external prostheses. For this, AgNPs were synthesized; physically, chemically, and functionally characterized, and incorporated into a polymer composite. The material obtained was assessed for certain physicomechanical properties, antimicrobial activity and cytotoxic effect.

## Materials and methods

**Fig. 1:**
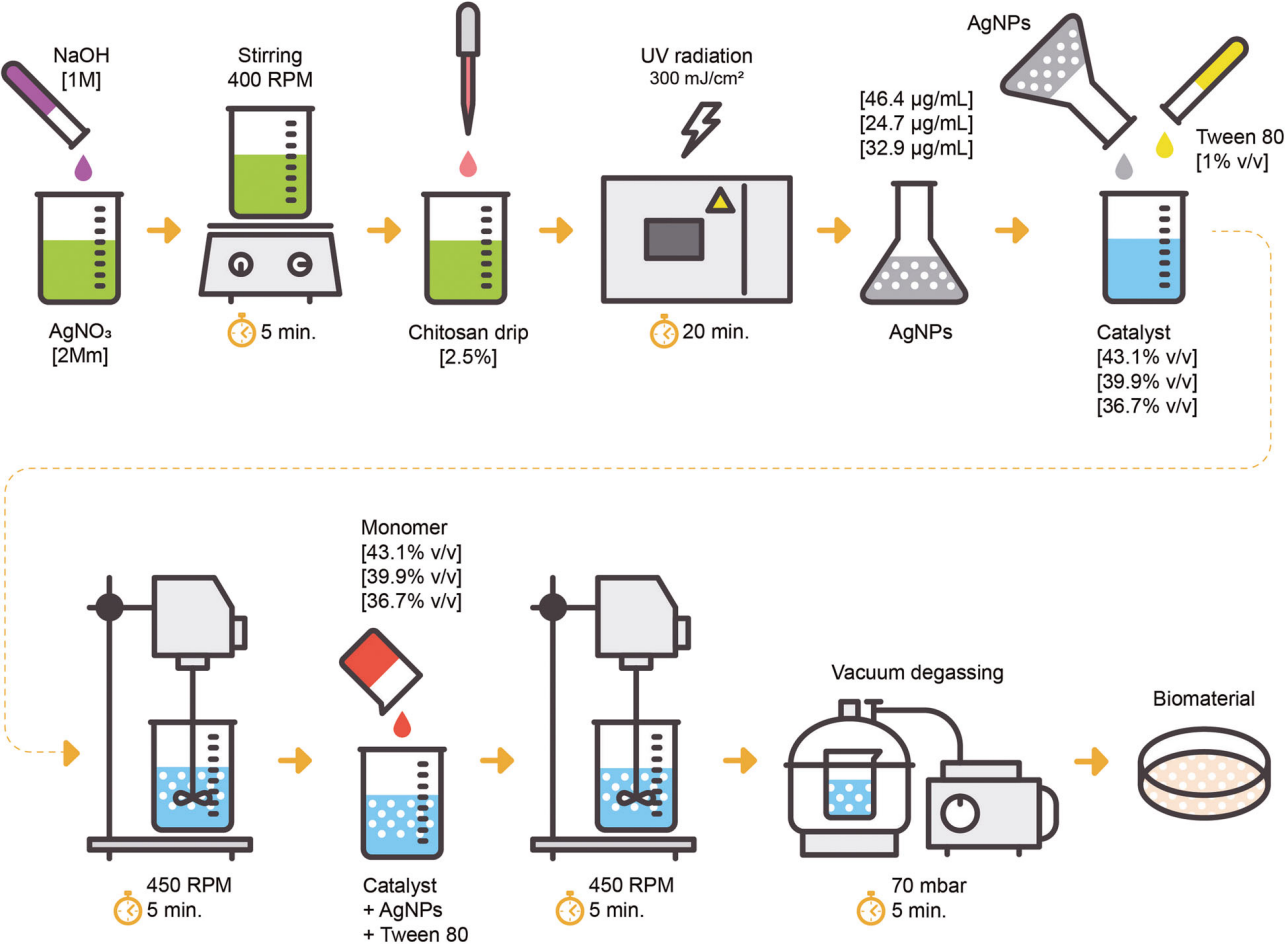
Schematic diagram of the synthesis of silver nanoparticle-containing polymer composite

A schematic diagram of the synthesis of a silver nanoparticle-containing polymer composite is depicted in Fig. 1.

### AgNPs synthesis

To prepare the AgNPs, silver nitrate (*AgNO*_3_) (PanReac Appli Chem CAS-No 7761-88-8), low-molecular-weight chitosan (SIGMA-ALDRICH CAS-No 9012-76-4), acetic acid (*CH*_3_*COOH*) (PanReac CAS-No 64-19-7), sodium hydroxide (NaOH) (SIGMA-ALDRICH CAS-No 1310-73-2), and type 1 water were used. All chemical reagents were of reactive analytical grade and were used without additional purification. The following AgNPs synthesis conditions were based on previous tests carried out by the authors: 100 mL of *AgNO*_3_ [2 Mm] was mixed with 200 *μ*L of NaOH [1 M] at 400 rpm for 5 min. Then, 33.3 mL of chitosan was dropped into acetic acid at 0.5%. Finally, the solution was subjected to the action of ultraviolet (UV) light radiation for 20 min at an intensity of 300 mJ/ (*cm*^2^). AgNPs obtained were diluted in type 1 water (diluent solution).

### AgNPs characterization

#### Physicochemical characterization

The obtained AgNPs were characterized for shape, morphology, and concentration. UV – Vis was employed to exhibit surface plasmon resonance (SPR) at wavelengths of 300 – 700 nm in a microplate spectrophotometer (MultiskanGo, Thermo Fisher Scientific Finland) [29].Total silver concentration was determined through atomic absorption spectrometry (AAS) (Thermo Scentific ICE 3000, USA) [30]. The hydrodynamic size of NPs was determined by dynamic light scattering (DLS) using Zetasizer Nano ZS. For this, NPs were diluted in type 1 water at a controlled temperature (23 ^∘^C) to obtain various concentrations at 0.10% volume. Three measurements were made, each with 30 balance seconds and 15 runs of 10 s [31]. Zeta potential of AgNPs was assessed using the same equipment, under the same measurement conditions and concentrations [32]. Finally, size and morphology were determined through transmission electron microscopy (TEM). For sample preparation, a 60 nm thick drop of each suspension was deposited on a carbon membrane (Tecnai F20 Super Twin TMP, FEI) [29].

#### Antimicrobial activity assessment

The antibacterial effect of AgNPs was assessed using the microdilution method against *Staphylococcus aureus* ATCC 25923 and methicillin-resistant *Staphylococcus aureus* (MRSA) [33]. Each bacterial strain was inoculated on Müller–Hinton agar (BD, REF 211438) and incubated for 24 h at 37 ^∘^C. A sample of each microorganism was cultured between 12 and 24 h in brain heart infusion (BHI; BD REF 211065) liquid medium at 37 ^∘^C. Turbidity of each bacterial suspension was adjusted to 0.5 McFarland in BHI medium. A spectrophotometer (Genesys 20, Thermo Scientific USA) was used to measure absorbance.

The microorganisms were incorporated into different AgNP dilutions in type 1 water [2.50 - 82.45 *μ*g/mL] to obtain a final concentration of 2.5*x*10^4^ UFC/mL in each dilution. These were stored at 37 ^∘^C in a shaking incubator (Rosy 1000, Thermolyne USA) at 75 rpm for 24 h in the dark.

The minimum bactericidal concentration (MBC) was assessed at 24 h to establish the antibacterial activity of AgNPs. For this, 10 *μ*L of each dilution was inoculated on Mueller–Hinton agar and incubated at 37 ^∘^C for 24 h; colony-forming units (CFU) were counted. Microorganism growth and sterility controls were performed at each trial. Each microorganism was assessed in triplicate.

### Composite formulation

The AgNPs obtained were employed to develop a nanostructured composite based on a commercial silicon elastomer matrix Ecoflex 00-50 (Smooth-On), which is a material certified under ISO 10993-10 [28]. The following preparation conditions for the composite were based on previous tests carried out by the authors: Elastomer samples were prepared with AgNPs at final concentrations of 16.49, 24.73, and 32.98 *μ*g/mL calculated based on concentration results by AAS. AgNPs were incorporated into the elastomer catalyzer (part B) via emptying, and 1% v/v polysorbate 80 (Tween 80) was added as a surfactant additive to facilitate the union between silicon and AgNPs. The parts were mixed for 3 min at 450 rpm using a mechanical agitator (IKA, RW 20 digital. Series RW 20). Finally, the elastomer monomer was incorporated (part A) at the same volume as part B and mixed for 5 min under the same conditions. The mixtures obtained were vacuum degassed using a desiccator and a vacuum pump (VACUUBRAND, ME 2C NT) at a pressure of 70 mbar for 5 min to extract air. Finally, the samples were curated for 3 h at room temperature (23.1 ^∘^C).

### Composite material characterization

Some physicomechanical properties, antimicrobial activiy and cytotoxic effect of the composite were characterized. The evaluation was performed using the material with and without AgNPs and with different concentrations of the diluent solution with AgNPs (type 1 water), as shown in Table 1. AgNP Samples were identified as “test samples” and non-AgNP sample as “control samples”. Each sample was prepared and assessed in triplicate to verify result reproducibility.

**Table 1:** Composites with different AgNP and AgNP diluent solution concentrations

		AgNPs	AgNPs diluent
Sample type	Name	concentration	solution
		(*μ*g/mL)	concentration (%)
AgNP samples (test sample)	S[16.49]	16.49	12.77
	S[24.73]	24.73	19.16
	S[32.98]	32.98	25.55
Non-AgNP samples (control samples)	S-C1[0]	0	12.77
	S-C2[0]	0	19.16
	S-C3[0]	0	25.55
	S-C4[0]	0	0

#### Evaluation of the physicomechanical characteristics of the composite

#### SEM-EDX analyses of the composite

A Scanning Electron Microscope (SEM) JEOL-JSM 6490LV was used for the study the presence and distribution of the AgNPs on the composite. Liquid nitrogen were used to freeze the samples and the fracture surfaces were observed. The composite with the highest concentration of AgNPs were also subjected to an energy dispersive X-ray analisis (EDX) accessory [34].

#### Fourier transform infrared spectroscopy (FTIR) of the composite

Characterization of the chemical composition was carried out using FTIR measurements with a PerkinElmer-Frotier (97668) spectrometer. The samples were cut into thin layers. All spectra were recorded in the range from 4000 to 400 cm-1 with a resolution of 1 cm-1. Also, each sample was scanned 32 times at 25 ^∘^C.

#### Evaluation of the density and mechanical characteristics of the composite

Tests were performed to assess hardness, density, compression, and tensile strength of the composite. These properties are assessed to study the behavior of the material when applying forces linked to prosthetic and orthotic interfaces.

Samples were submitted to a standard hardness test as per the international standard ASTM D 2240-15 using a Shore A (0-100 HA, REF.MDT 10 A) portable durometer and performing five hardness measurements at different sample positions [35]. Sample density was assessed following the procedures established by the international standard ASTM D 792-13, method B [36]. From each sample, four test specimens were obtained in the form of sheets measuring 20 x 20 x 3 *mm*^3^. In addition, a dissymmetric scale (Shimadzu Medical Systems, model AX200), a stationary support, an immersion recipient with ethanol 70% (*ρ* = 0.86 g/mL), and a wire sample holder were employed. Specimen mass in air was determined on the sample holder, and then specimens were dipped in ethanol to determine composite density. Finally, the density values obtained from each sample were averaged. The test procedure was conducted at a temperature of 23 ±2 ^∘^C and a relative humidity of 50% ±10%.

Furthermore, sample compression strength was assessed based on the international standard ASTM D395-16^a^ (2016), method B [37]. From each sample, three cylindrical test tubes of 29 ±0.1 mm diameter and 11.6 ±0.5 mm thickness were obtained. Samples were placed between two rigid compression steel plates and subjected to gradual compression until reaching 25% of the original thickness for each sample, using a 5582 INSTRON universal trial machine. The procedure was performed at a speed of 250 mm/min, temperature of 23 ±2 ^∘^C and relative humidity of 50% ±10%. The total load applied to the samples was 800 N. The compressed samples were stored in a UN55 Memmert universal kiln at 70 ^∘^C for 22 h. Samples were cooled at room temperature for 30 min, and their final thickness was measured with a digital gauge with 0.01-mm accuracy. Compression was calculated as using Eq. 1:

1 $$\begin{array}{@{}rcl@{}} C\% = \left[\left(t_{0} - t_{l}\right) / ((t_{0} - t_{s})\right]\;x\;100 \end{array} $$

Where *t*_0_ is the original sample thickness; *t*_*l*_ is the final sample thickness, and ts is the thickness between the compression plates.

Finally, a tensile test was performed to assess tensile yield strength, rupture strength, and modulus of elasticity as per the international standard ASTM D 412-15 (2015), method A [38] using a dumbbell test tube. Five type-D samples were produced for each specimen. For this, a 200 x 220 x 3 *mm*^3^ sheets were obtained from the material by injection from an acrylic mold. To obtain the test tubes, a manual cutting machine was used. For these purposes, a 5582 INSTRON universal trial machine was used. An INSTRON load cell with a 1 kN load capacity and pneumatic clamping jaws were employed. Trial speed was of 250 mm/min. The room temperature during the trial was 23 ^∘^C and the relative humidity was 50%. To calculate tensile strength (*σ*u) and maximum elongation (*λ*u), Eqs. 2 and 3 were used, respectively:

2 $$\begin{array}{@{}rcl@{}} \sigma u = F_{max}\;/\;A\;; ~ where~ A = w\;x\;t \end{array} $$

3 $$\begin{array}{@{}rcl@{}} \lambda u= l_{max}\;/\;l_{0} \end{array} $$

Where *F*_*max*_ is the maximum strength, and A is the initial area of the calibrated zone transversal section calculated by multiplying the width (w) and thickness (t) of the test tube in mm. *l*_*max*_ represents the maximum test tube length and *l*_0_ the initial calibration length.

#### Evaluation of the antimicrobial activity of the composite

**Fig. 2:**
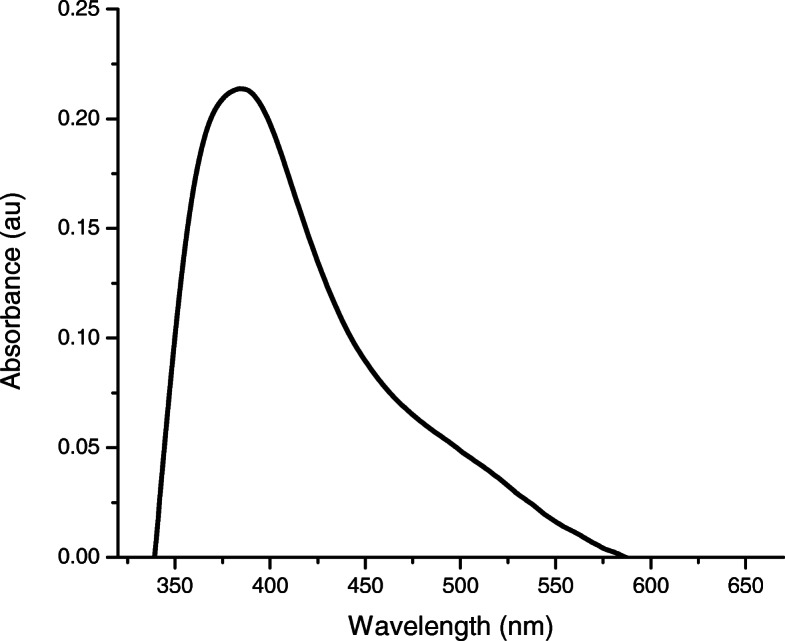
UV–Vis spectroscopy of AgNPs reduced by chitosan

The provisions from international standard ISO 22196:2011 were followed and Fig. 2 shows a diagram of this process [39]. The evaluation was performed gainst *Staphylococcus aureus* ATCC 25923 and MRSA, which are gram-positive bacteria frequently found on the skin [40]. With each concentration, three sheets of the material measuring 50 x 50 x 3 *mm*^3^ were obtained. All samples were sterilized with ethanol (70%), and subjected to UV radiation for 20 min. Each bacterial strain was inoculated on Müeller–Hinton agar for 24 h at 37 ^∘^C under aerobic environment and cultured for 24 h in BHI medium at 37 ^∘^C. Turbidity of each suspension was adjusted to 0.5 McFarland in BHI, and the density of bacteria was diluted to 1x 10^4^ UFC/mL. A UV–Vis spectrometer (UV-Vis) (MultiskanGo, Thermo Fisher Scientific Finland) was used for measuring the optical density at 600 nm. Next, 100 *μ*L of the bacterial suspension was added on each sample and covered with a 40 x 40 *mm*^2^ sterile high-density polypropylene square film (n = 72). Each sample was incubated at 37 ^∘^C for 24 h. Samples were washed with 50 mL of an isotonic saline solution. Ten serial dilutions were made in 500 *μ*L of BHI medium based on a primary dilution with 500 *μ*L of the microorganism wash. Then, 10 *μ*L were extracted from the final dilution and seeded into Petri plates with Mueller-Hinton agar and incubated for 24 h at 37 ^∘^C. Finally, the number of visible colonies in the Petri plates was counted, and antimicrobial activity (R) was calculated using the following Eq. 4:

4 $$\begin{array}{@{}rcl@{}} R = U_{t} - A_{t} \end{array} $$

Where *U*_*t*_: is the decimal logarithm average for the total of viable colonies recovered from the samples without AgNPs after 24 h (cells/ *cm*^2^); *A*_*t*_: is the decimal logarithm mean for the total of viable colonies recovered from the samples with AgNP after 24 h (cells/ *cm*^2^). The R value of ≥2.00 was considered equivalent to the elimination 99% bacteria [41].

#### Evaluation of cell viability in the composite

The cytotoxic effect of the material was evaluated by cell viability in human epidermal keratinocytes (HaCaT). It was used a lactate dehydrogenase (LDH) detection technique [42]. For this, samples were prepared in FalconⓇ 24-wells cell culture plates in triplicate following the preparation conditions described in the composite formulation at 25 ^∘^C. The cells were cultivated on samples surface in culture medium Dulbecco’s Modified Eagle Medium (DMEM) supplemented with 10% fetal bovine serum (FBS). After 12 h exposure, Kit RayBioⓇ LDH-Cytotoxicity Assay was used. Cells were submitted to 50 *μ*L of lysis solution for 3 h as positive control and untrated cells were used as negative control. 10 *μ*L of supernatant cell were transferred to 96-wells cell culture plates, then 100 *μ*L of LDH was added to each sample for 30 minutes. Then, the absorbance was measured at 450 nm using a spectrophotometer (Thermo Scientific Multiskan Go) at 25 ^∘^C and the percentage of cell viability was calculated with the following equation:

5 $$ {{}\begin{aligned} Viability \,=\, 100\!- (((OD test sample - OD negative control))\\ /((OD positive control - OD negative control)) x 100\%) \end{aligned}}  $$

Where “OD tests sample” is the optical density of each sample evaluated, “OD negative control” is the optical density of untreated cells, and “OD positive control” is the optical density of lysate cells after 3 h.

**Statistical analysis**

All data obtained from the mechanical tests of the test samples were compared to those of the control samples through multifunctional analysis of variance (ANOVA), with each mechanical property as the dependent variable and the presence and concentration of AgNPs as independent factors. Statistical analyses were performed using the Statgraphics Centurion XV software. P < 0.05 values were considered statistically significant (at 95% reliability).

## Results

### AgNPs synthesis and physicochemical characterization

**Fig. 3:**
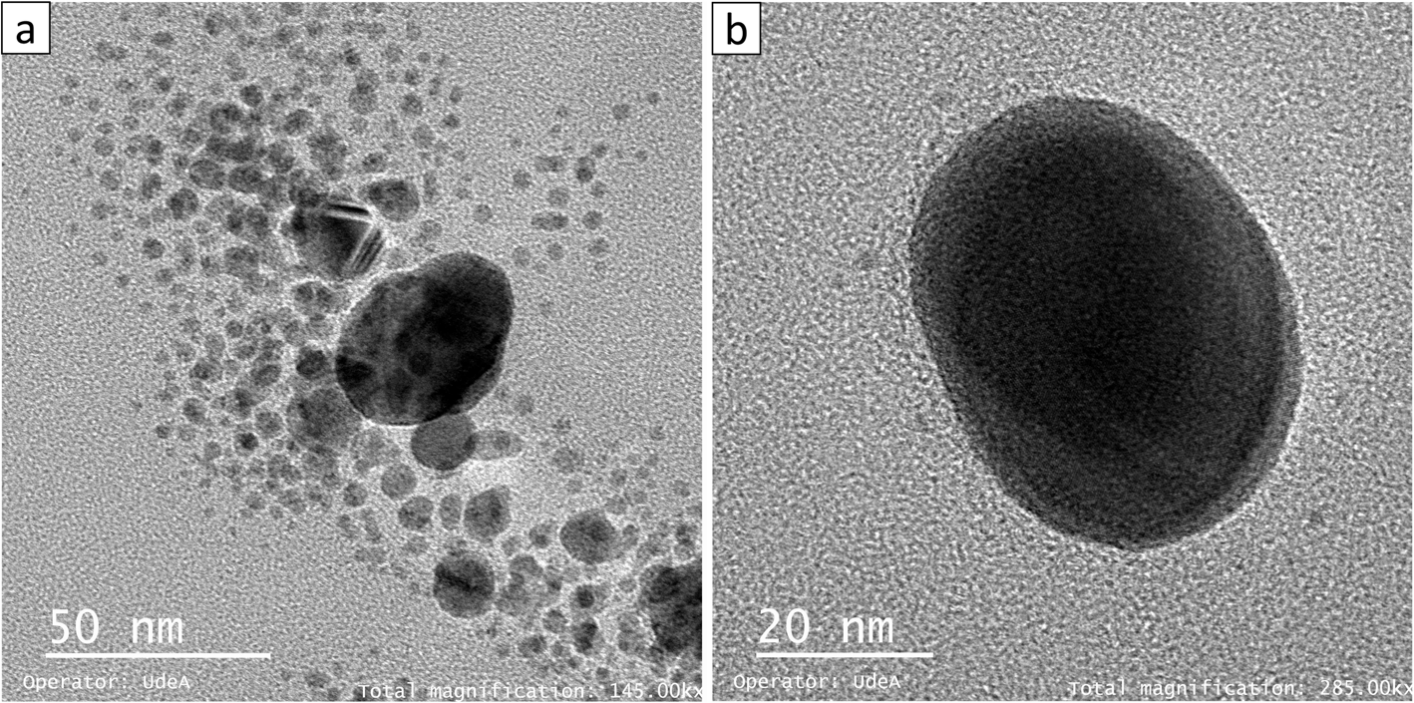
Micrograph of AgNPs conducted through TEM **a** 50 nm magnification, **b** 20 nm magnification

**Fig. 4:**
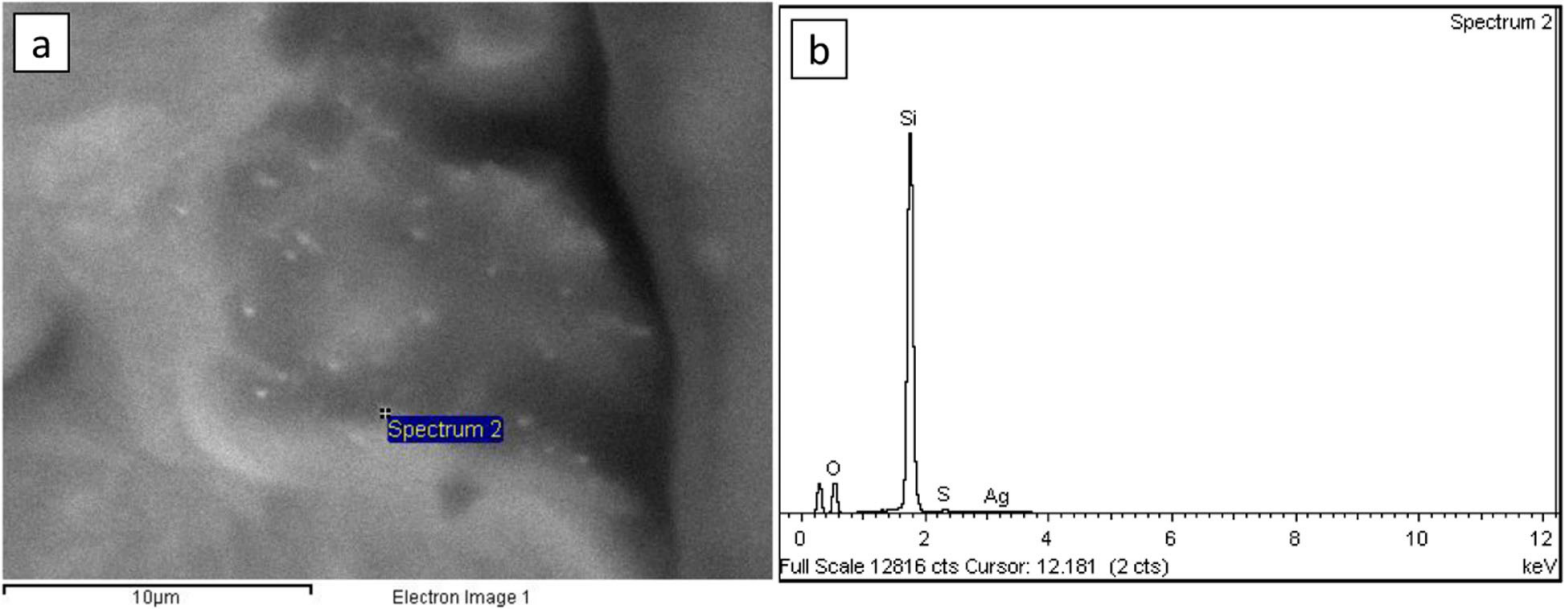
SEM-EDX analyses of the composite. **a** distribution of AgNPs on the composite, **b** EDX spectrum of the composite with AgNPs

A light gray colloidal AgNPs solution was obtained. RSP ranging from 320 to 420 nm, with a pronounced peak of maximum absorbance at 390 nm, was noted in UV–Vis spectroscopy, indicating the formation of AgNPs measuring 5–50 nm, as inferred by wavelength width [43]. Additionally, TEM revealed that AgNPs were spherical. Figures 3 and 4 shows the absorbance spectrum and a micrograph of AgNPs.

#### Physicochemical properties of AgNPs

The results of the physicochemical characterization are presented in Table 2.

**Table 2:** Characterization of physicochemical properties of AgNPs

Characteristic	Value
AgNP concentration (AAS)	129 ±12.30 *μ*g/mL
Average hydrodynamic diameter (DLS)	130.2 ±63.6 nm
Zeta Potential (DLS)	5.74 ±3.86 mV

#### Antimicrobial effects of AgNPs

The MIC of AgNPs against *Staphylococcus aureus* ATCC 25923 and MRSA was 16.49 *μ*g/mL. The growth and sterility controls conducted were appropriate for all assays.

### Composite characterization

#### SEM-EDX analyses of the composite

**Fig. 5:**
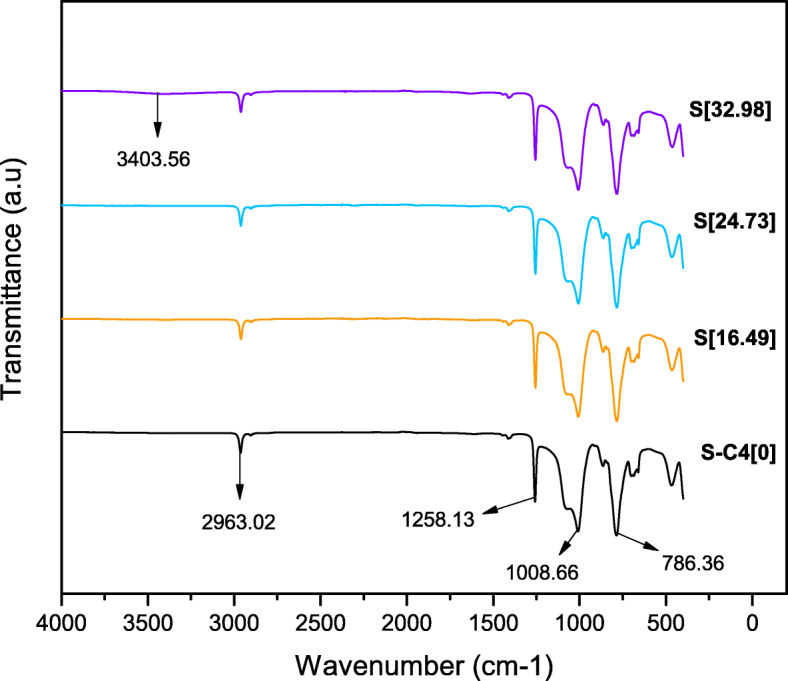
FTIR spectrum of the composite with different concentrations of AgNPs

A low density of AgNPs confirmed with the presence of Ag was observed in Fig. 5. A relatively polydispersed AgNP was shown with no agglomeration in the compound. Furthermore, the figure shows the chemical composition of the samples, obtained from the EDS spectra. The existence of sulfur (0.84%), oxygen (30.07%), silver (1.36%) and silicon (67.74%) in the composite samples was found. No other relevant compounds were identified.

#### Fourier transform infrared spectroscopy (FTIR) of the composite

**Fig. 6:**
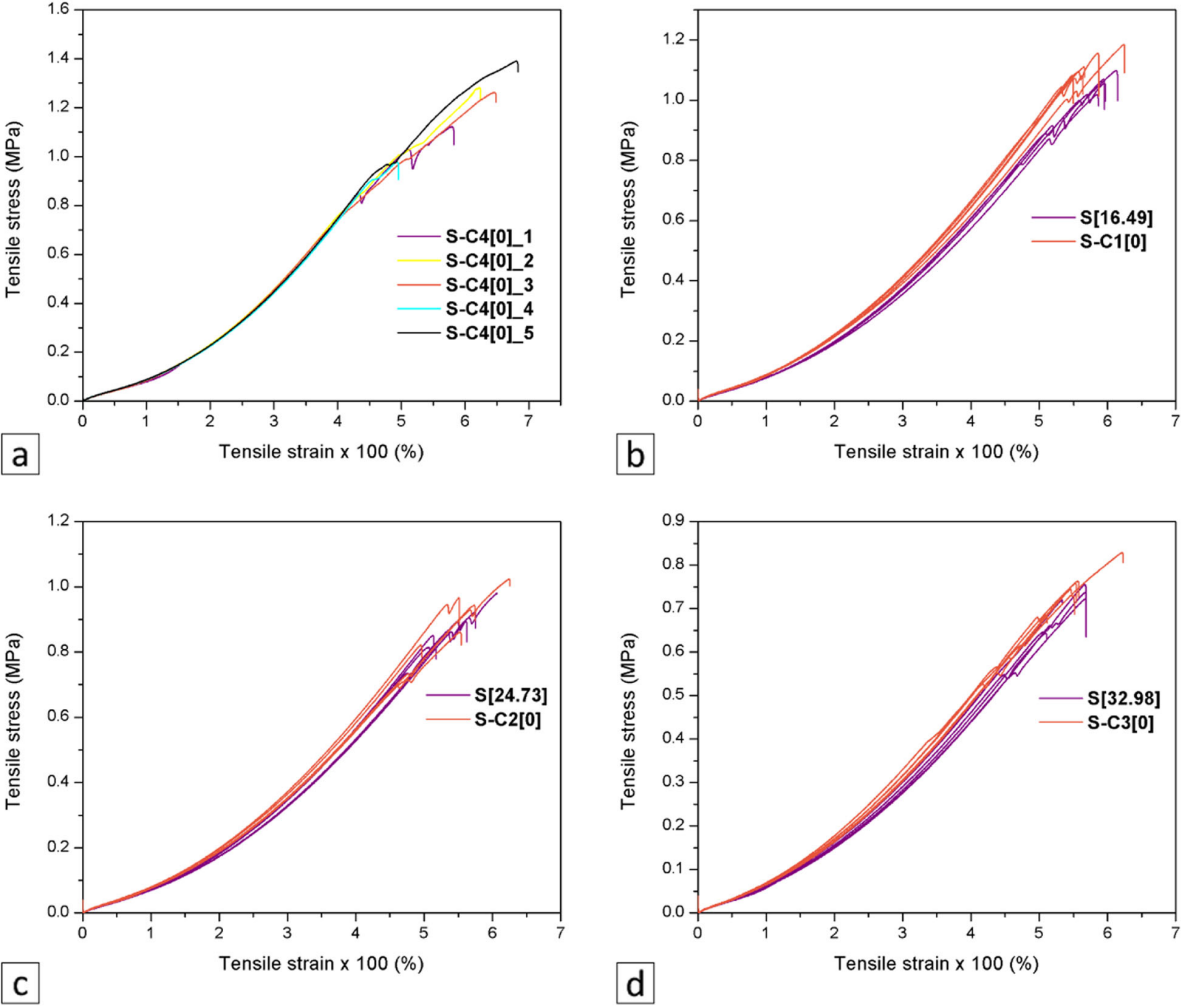
Tensile stress-strain curves of **a** S-C4[0]_1 to SC4[ 0]_5, **b** [16.49] and S-C1[0], **c** S[24.73] and S-C2[0], **d** S[32.98] and S-C3[0]

The FTIR spectra from the samples exhibited characteristic IR bands from PDMS [44, 45]. Figure 6 shows the results obtained. Peaks were found at 2963 cm-1 (CH3 stretching in methyl groups), 1248 cm-1 (Si-CH3 bending), 1071 cm-1 and 1008 cm-1 (Si-O-Si asymmetric stretching), and 786 cm-1 (Si-CH3 bending). However, only in the highest concentration of AgNPs, a band with 97.09% trasmittion was observed in 3403.56 cm-1, related to the presence of water as diluent solution of the nanoparticles.

#### Density and mechanical characterization of the composite

Table 3 presents average Shore A hardness, density, percentage of deformation by compression, ultimate tensile strength, rupture strength, and modulus of elasticity of the samples prepared as described in Table 1.

**Table 3:** Physicomechanical properties of the composite with different concentrations of AgNPs (*μ*g/mL) and AgNP diluent solution

Sample type	Sample	AgNPs	Average	Average	Maximum	Ultimate	Tensile	Tensile	Break
	name	concentration	hardness	density	deformation by	tensile	rupture	elasticity	elongation
		(*μ*g/mL)	(Shore A)	(g/cm3)	compression (%)	strength (MPa)	strength (MPa)	modulus (MPa)	(%)
AgNP	S[16.49]	16.49	4.8 ±0.27	1.020 ±0.00	10.46 ±0.07	1.10 ±0.03	1.06 ±0.03	0.30 ±0.01	597.70 ±11.80
samples	S[24.73]	24.73	3.6 ±0.22	1.022 ±0.00	18.39 ±0.05	0.90 ±0.06	0.89 ±0.06	0.20 ±0.01	555.10 ±39.74
(test samples)	S[32.98]	32.98	3.4 ±0.41	1.013 ±0.00	17.12 ±0.19	0.70 ±0.04	0.71 ±0.04	0.20 ±0.01	549.50 ±26.54
Non-AgNP	S-C1[0]	0	3.8 ±0.57	1.024 ±0.00	14.48 ±0.07	1.10 ±0.05	1.12 ±0.05	0.30 ±0.01	578.0 ±29.26
samples	S-C2[0]	0	3.4 ±0.41	1.022 ±0.00	11.60 ±0.06	0.90 ±0.08	0.92 ±0.08	0.20 ±0.02	560.20 ±46.63
(control	S-C3[0]	0	3.7 ±0.27	1.016 ±0.00	25.17 ±0.16	0.80 ±0.05	0.75 ±0.05	0.20 ±0.01	559.10 ±39.94
samples)	S-C4[0]	0	4.4 ±0.22	1.022 ±0.00	13.44 ±0.08	1.20 ±0.16	1.20 ±0.16	0.30 ±0.02	606.20 ±72.19

**Fig. 7:**
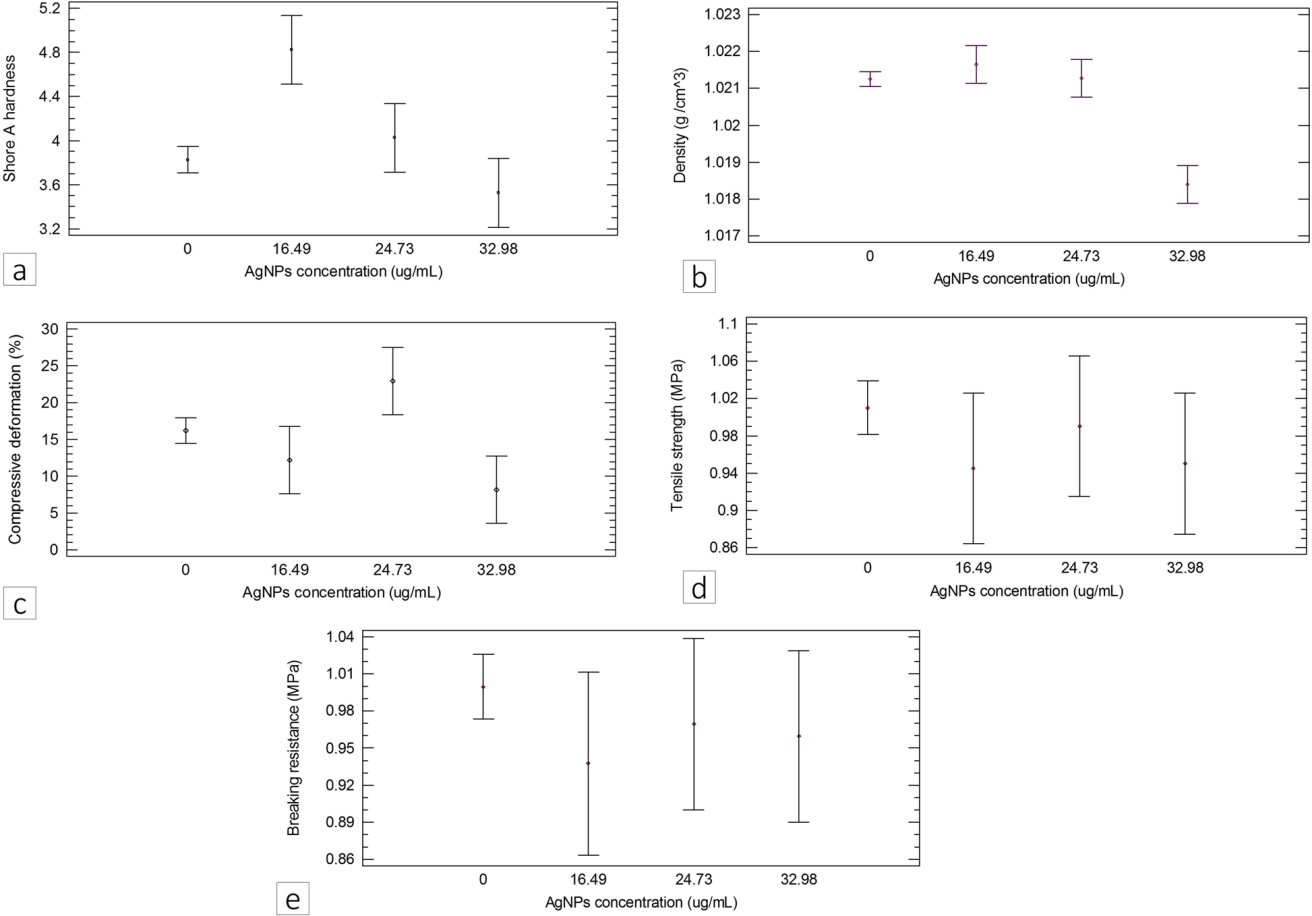
Graphs comparing physicomechanical properties of the composites with different AgNPs concentrations **a** Hardness, **b** Density, **c** Deformation by compression, **d** Tensile strength, **e** Rupture strength

**Fig. 8:**
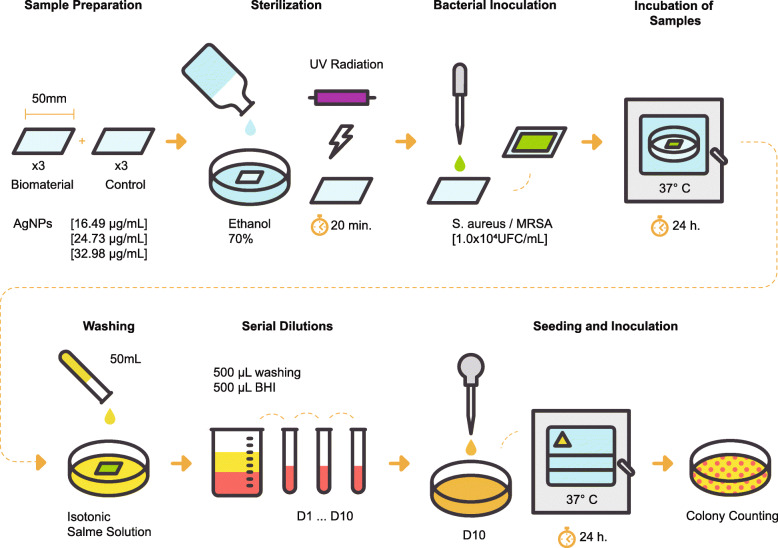
Diagram of the antimicrobial activity evaluation on the composite

Figure 7 presents the tensile stress curves of each sample, and Fig. 8 shows the average values of hardness, density, deformation by compression, tensile strength, and tensile rupture strength of the composite at different AgNP concentrations, as well as results of comparison between the test and control samples using multifactorial ANOVA with 95% reliability. The samples with an AgNP concentration of 16.49 *μ*g/mL showed a greater Shore A hardness than the samples with AgNP concentrations of 0, 24.73, and 32.98 *μ*g/mL, although the differences were not statistically significant (P >0.05). In contrast, the samples prepared using 32.98 *μ*g/mL AgNPs showed a significantly lower density than those prepared using 0, 16.49 and 24.73 *μ*g/mL AgNPs. In terms of deformation by compression, there were no significant differences between the control and test samples with 16.49 and 24.73 *μ*g/mL AgNPs as well as between samples with 16.49 and 32.98 *μ*g/mL AgNPs. Finally, there were no statistically significant differences (P >0.05) in tensile strength, rupture strength, and modulus of elasticity among samples with different AgNPs concentrations.

#### Antimicrobial activity of the composite at the surface level

**Fig. 9:**
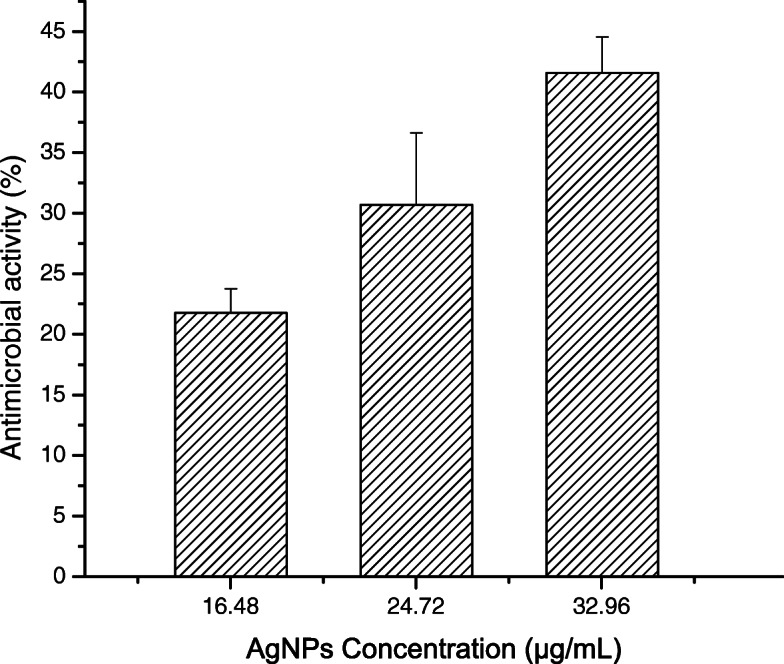
Antimicrobial activity of the AgNP composite against *Staphylococcus aureus* ATCC 25923

**Fig. 10:**
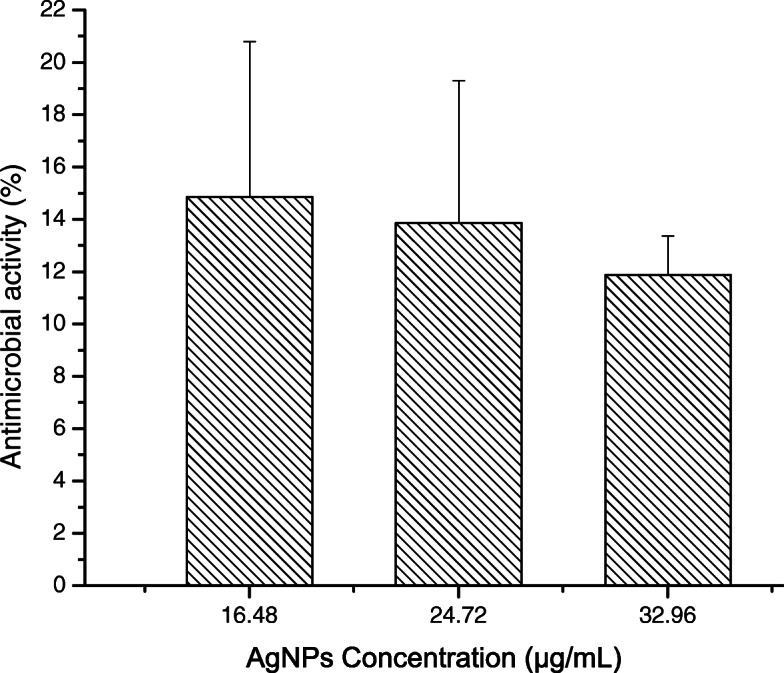
Antimicrobial activity of the AgNP composite against methicillin-resistant *Staphyloccocus aureus* (MRSA)

Figures 9 and 10 display the antimicrobial activity of the composite at increasing concentrations of AgNPs against *Staphylococcus aureus* ATCC 25923 and MRSA, respectively. The microbial activity of the AgNP composite at 32.98 *μ*g/mL (41.58 ±2.97%) was higher than that at 16.49 *μ*g/mL (21.78 ±1.98%) against *Staphylococcus aureus* ATCC 25923. However, there were no significant differences (P >0.05) in antimicrobial activity between samples with concentrations of 16.49 (14.85 ±5.94%), 24.73 (13.86 ±5.44%), and 32.98 *μ*g/mL (11.88 ±1.48%) against MRSA. In addition, the antimicrobial activity of AgNP concentration of 24.49 *μ*g/mL against *Staphylococcus aureus* ATCC 25923 was higher than that of and AgNP concentration of 32.98 *μ*g/mL against MRSA.

#### Evaluation of cell viability in the composite

**Fig. 11:**
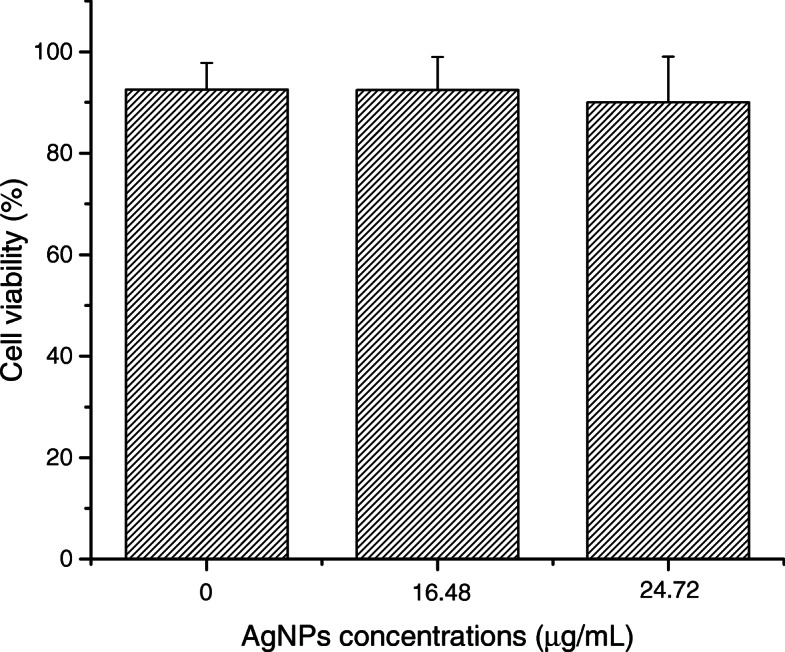
Cytotoxicity graphs from the MTT assay for composite samples with different concentrations of AgNPs

The results of the lactate dehydrogenase detection test in human keratinocytes showed that in all the samples evaluated with different concentrations of AgNPs, the cell viability was equal to or greater than 90% after 12 h of exposure (Fig. 11). It was no found significant differences between the percentages of cell viability in samples with different concentrations of AgNPs, therefore, dependence of cytotoxicity on AgNPs concentration cannot be determined for this case.

## Discussion

This article presents innovations in the processing and characterization of a composite AgNP material for application as the interface for external prostheses, with abilities to reduce *Staphylococcus aureus* and MRSA proliferation.

To this end, AgNPs were synthesized through a photochemical method, described in the methodology, using chitosan as a reducing agent. The NPs obtained were confirmed to be on Ag based on SPR assessed through UV-Vis [29]. The bands observed were wide, suggesting a different size distribution of NPs. Moreover, the UV plasmon of AgNPs has been found to exhibit peak absorption on the range of about 400-500 nm which depended to size, shape and distribution as found in this study [46]. The Fig. 3 also shows an hump in UV spectra around 475-525 nm. This may be related to syntheses from natural sources tend to present two or three populations with different proportions and the presence of NaOH in the synthesis [47]. This correlates with this work and Fig. 4.

During synthesis, UV radiation was delivered to break chitosan bonds. NPs were formed thanks to the capacity of the pair of electrons of nitrogen atoms –NH2 present in the chitosan to form complexes with Ag ions, thus generating a chelation effect [48]. The formation of AgNPs was influenced by wavelength, power, energy, and UV irradiation time, in addition to the presence of chitosan during *AgNO*_3_ reduction [49]. Chitosan is an efficient reducer in UV radiation-assisted production of AgNPs [50].

The AgNP concentration could be determined through AAS, and a positive surface zeta potential was observed due to the power of NPs themselves as well as their interaction with chitosan, which charged molecules positevely [51, 52]. Consistent with previous reports [12, 29, 31], spherical and semi-spherical NPs were obtained. Previous studies have reported antimicrobial activities of spherical NPs. This activity is usually associated with size, oxidation capacity, and release of Ag ions from NPs [12, 53]. Hence, AgNPs present antimicrobial activity probably due to their size and their ion releasing capacity. In contrast, the toxic effects of AgNPs on microorganisms may increase in the presence of chitosan as a reducing agent. However, Wen Lou et al. [48] have reported lower antimicrobial activity of chitosan alone than that of AgNPs.

The results of antimicrobial effects of AgNPs against *Staphylococcus aureus* ATCC 25923 and MRSA in this study were consistent with those in previous studies [54, 55]. This may be attributed to the synthesis method as well as the composition and physicochemical properties of AgNP, which allowed for the electrostatic interaction of NPs with microorganisms, to permeate the cells through peptidoglycans in cell walls and induce damage, which may trigger cell death [56].

From the AgNPs obtained, nanostructured composites were manufactured using Ecoflex 00-50Ⓡ, a commercial silicone elastomer matrix, with different concentrations of AgNPs (0, 16.49, 24.73, and 32.98 *μ*g/mL) and type 1 water (0, 12.77,19.16, and 25,55%), in accordance with the methodology described. A SEM micrograph of the composite with AgNP allowed to observe the nanostructures in the material. In addition, sulfur, oxygen, silver, and silicone were found in the samples through EDS analysis. The oxygen identified may be related to the chamber of the accessory and the silicone elastomer matrix. While the sulfur identified, it has been found that it can arise from the presence of AgNPs in biosolids [57]. However, this presence is minimal.

A FTIR analysis was carried out to study chemical composition of the samples. Characteristic bands of PDMS were found due to this is the higher component of silicones, such as that used as the matrix of the composite [25]. On the other hand, only in the samples with the highest concentration of AgNPs a band could be observed at 3403.56 cm-1. This is related to the presence of H-O-H signals that can appear in wet samples, between approximately 3600 and 3100 cm-1 [58]. Thus, since the samples with the largest nanoparticles were diluted in higher water concentration, this could be observed.

Conversely, literature has described that NPs may change the mechanical properties of the materials to which they are added. Thus, we assessed some mechanical properties of the polymer material obtained using various AgNP concentrations. In addition, the mechanical characteristics of the material must be determined as they provide an idea of the expected performance of the interface in the application for lower limb orthotic and prosthetic devices, given that these are subjected to internal loads and stresses caused by patient gait and weight [59, 60].

Several authors have found that increased concentration of NPs in a matrix may increase the material’s mechanical properties [61]. This study showed that composites with higher AgNP concentrations were less deformed by compression than samples without AgNPs. This phenomenon can be explained by the role that NPs may have as nanoreinforcers to improve the mechanical properties of the new materials, as NPs become anchorage points, preventing the relative sliding between chains and thus strengthening the material.

Furthermore, similar to that in other studies [62], no statistically significant differences were found in this study in terms of Shore A hardness, tensile strength, rupture strength, and modulus of elasticity between samples prepared without AgNPs and those with the highest AgNP concentration. The underlying reason for this may be that AgNPs did not affect the formation of the polymer network or the bonding of silicon elastomer molecules. NPs may have been tightly bonded to the matrix. Alternatively, given that AgNPs concentrations assessed were really low compared to the concentration of the polymer to which they were added, they were not capable of reinforcing the mechanical properties of the material.

However, samples with high AgNPs concentrations assessed showed much lower density than non-AgNP Samples, mostly due to the fact that the higher concentration of NPs may have affected the crosslinking of the matrix polymer chains, which impacted the compaction degree of the material and its ultimate compression stress. On the other hand, samples prepared with the highest AgNPs diluent solution presented lower mechanical properties than those prepared without AgNPs. These were the result of the decrease in the concentration of the polymer in materials due to the water volume required for each concentration, that is, as the concentration of the AgNPs diluent solution increases, the matrix concentration should decrease. This may lead to a reduction in the bonding forces of monomers in material polymerization or a decrease in the monomer amount which reacts and affects the catalytic process of the polymer [63, 64].

Similarly, Mancuso has discovered that silicone additives may reduce their mechanical properties due to the presence of water [65]. Water in polymer solutions may reduce the bonding forces of monomers during the polymerization process, which is reflected in structural changes and the polymer’s plastic deformation resistance [64]. Also, the immiscibility of the Ecoflex 00-50 Ⓡwith type 1 water, where AgNPs were found, may act as plasticizer and alter the mechanical properties of the material.

The mechanical results presented in this study may not generate any inconvenience, as the values obtained were within the acceptable ranges of hardness (0 - 20 on the Shore A scale) [66], density (1.0 - 2.5 g/cm^3^) [67], deformation by compression (8 - 59%) [68], ultimate tensile, and rupture strength (0.030 - 0.248 MPa) [69] and modulus of elasticity (0.124 - 3.45 MPa) [70], for lower limb orthotic and prosthetic devices.

On the other hand, all AgNP composites showed antimicrobial activities against *Staphylococcus aureus* ATCC 25923 and MRSA. AgNPs reduce the growth of microorganisms that cause skin infections in patients using external lower limb prostheses and orthoses. Hence, the developed composites inhibited the proliferation of microorganisms due to the presence of AgNPs. Nevertheless, the bacteria assessed were not completely removed.

In this study, NPs were introduced in a silicone elastomer polymer matrix and acted as Ag ion deposits, releasing the ions that acted against microorganisms. However, the matrix showed evidently reduced releasing capacity of Ag ions compared to the antimicrobial activity of AgNP suspension at a concentration of 16.49 *μ*g/mL, which produced a bactericidal effect against the two microorganisms assessed.

The greatest antimicrobial activity against *Staphylococcus aureus* ATCC 25923 was noted in test samples with the highest AgNP concentration (32.98 *μ*g/mL). Increase in antimicrobial activity against Staphylococcus aureus from samples with the highest concentrations of AgNPs assessed may be due to increased release of Ag ions resulting from a higher number of NPs in the material. Therefore, a higher concentration of AgNPs would allow for total elimination of the microorganism, although the maximum limits of concentrations acceptable for AgNPs to avoid cytotoxic effects must also be considered [71]. The results obtained in this work are consistent with those in other studies [72], showing that a greater release of Ag ions may increase the antimicrobial effects of NPs against microorganisms.

Conversely, no statistically significant differences were found in the antimicrobial activity against MRSA among composites with 16.49, 24.73, and 32.98 *μ*g/mL AgNPs. In addition, the antimicrobial activity against *Staphylococcus aureus* ATCC 25923 was higher than that against MRSA in the case of the test samples with AgNP concentrations of 24.49 and 32.98 *μ*g/mL.

Literature has proven that MRSA shows greater virulence than *Staphylococcus aureus* [73]. The virulence factors are molecules produced by bacteria, established in their genome, which allow them to survive under extreme conditions in the host [74]. Anisha et al. [73] found that antimicrobial effect against *Staphylococcus aureus* was greater than that against MRSA in a sponge composed of chitosan and AgNPs, as reported in the present study. The authors decided to assess whether virulence prevalence was higher in MRSA strains than in *Staphylococcus aureus* and found that all MRSA strains showed the presence of staphyloxanthin, which is a virulence factor acting as an antioxidant [75]. This factor allows the microorganism to detoxify reactive oxygen species (ROS) [76]. This way, the a greater amount of staphyloxanthin present in MRSA helps it fight the free radicals produced by AgNPs, thus increasing the strength of microorganisms against NPs [73].

The effects from Tween 80 on bacteria have also been studied. Tween 80 has been used as an additive to ease the bond between the matrix and AgNPs. This surfactant may increase the permeability of bacterial cells and improve the antimicrobial activity of the agents to which they are added, such as antibiotics [77]. Nonetheless, this study has not assessed the effect of this surfactant on the microorganisms assessed as all the samples of the material studied, including controls, were prepared using the same Tween 80 concentration (1% in volume).

Studies claim that AgNPs have low cytotoxicity at low concentrations and would be suitable for biomedical applications [78, 79]. Nevertheless, the design and manufacture of new biomaterials with the purpose of use in humans require a biocompatibility evaluation [80–82]. In this study, the biomaterial developed with the different concentrations of AgNPs assessed does not cause the death of a large cell population and could maintain cell viability for 12 h. Some authors have suggested that using polymers with silver nanoparticles, either as matrix or coatings, improve material biocompatibility [82]. Besides investigations have concluded that AgNPs immersed in a polymeric matrix may decrease the cytotoxic effect compared to these immersed in an aqueous solution [83]. Thus, it is possible that in this study, the polymeric matrix has prevented significant damage to the membrane of the HaCaT cells evaluated by avoiding direct contact of the AgNPs with these cells.

This study helped develop a promising AgNP polymer composite with AgNPs for the design of lower limb external orthotic and prosthetic interfaces that would contribute to decrease the proliferation of several microorganisms in the contact areas between the patient’s skin and the devices. Nevertheless, further antimicrobial studies on other microbiological strains are required, as well as to continue studying cell viability and the cytotoxic and genotoxic effects of the material obtained given the aim of its application.

## Conclusion

This article discussed the processing and characterizing of an AgNP silicone elastomer composite. To this end, AgNPs were synthesized through an *AgNO*_3_ photochemical reduction method using chitosan. NPs were physico-chemically characterized, detecting spherical and semi-spherical shapes. The AgNPs allowed for the complete inhibition of *Staphylococcus aureus* ATCC 25923 and MRSA.

It was possible to incorporate the AgNPs in a silicone elastomer, finding that the composite developed presented antimicrobial activity against *Staphylococcus aureus* ATCC 25923 and MRSA when compared to nonAgNP material samples. The microorganism reduction was possibly due to the presence of NPs; however, there were no signs of the total elimination of the bacteria assessed.

The growth reducing capability of *Staphylococcus aureus* ATCC 25923 presented by the composite material with the highest AgNPs concentration assessed was higher than in MRSA by roughly 29.70%. This is the result of the greater presence of virulence factors in antibiotic-resistant strains, which act as antioxidants against the free radicals produced by AgNPs.

Furthermore, the AgNP material exhibited ultimate tensile strength, tensile rupture strength and tensile elasticity modulus from 0.70 to 1.10 MPa, 0.71 to 1.06 MPa and 0.20 to 0.30 MPa, respectively. The mechanical properties of the composite were within the acceptable conditions for its use as lower limb external orthotic and prosthetic interface in accordance with current commercial values and the values reported in the literature.

In addition, acording to the results obtained from cell viability, the biomaterial could be used as an external biomedical device that have contact with the skin. Nevertheless, it is necessary to carry out other cytotoxic tests such as irritability, allergy, genotoxicity, and mutagenicity.

This research allowed the development of a polymer composite containing AgNPs, which is a promising material for the design of lower limb external orthotic and prosthetic interfaces as it may help reduce the proliferation of *Staphylococcus aureus* ATCC 25923 in contact areas.

## Data Availability

All data generated or analysed during this study are included in this published article.

## Abbreviations

AAS: Atomic absorption spectroscopy
AgNPs: Silver nanoparticles
ANOVA: Analysis of variance
ATCC: American type culture collection
ATP: Adenosine triphosphate
BHI: Brain heart infusion
CFU: Colony Forming Units
DLS: Dynamic light scattering
DMEM: Dulbecco’s Modified Eagle Medium
DNA: Deoxyribonucleic acid
FBS: Fetal bovine serum
IR: Spectroscopy Infrared spectroscopy
LDH: Lactate dehydrogenase
MBC: Minimum bactericidal concentration
MIC: Minimum inhibitory concentration
MRSA: Methicillin-resistant *Staphyloccocus aureus*
R: Antimicrobial activity of the materials
RPM: Revolutions per minute
SPR: Surface plasmon resonance
Tween 80: Polysorbate 80
TEM: Transmission electron microscopy
UV-Vis: Ultraviolet- visible spectroscopy
